# A systematic review of school‐based interventions to promote digital resilience in children

**DOI:** 10.1111/bjdp.70038

**Published:** 2026-02-18

**Authors:** Georgina C. Halliwell, Sarah E. Rose, Richard Cooke, Daniel Herron

**Affiliations:** ^1^ University of Staffordshire Stoke‐on‐Trent UK

**Keywords:** behaviour change, children, classroom, digital resilience, online

## Abstract

This systematic review investigates the novel concept of digital resilience, how school‐based interventions address this and the extent to which they are effective. Four databases were searched and 15 interventions meeting the inclusion criteria were identified. Quality was assessed using the Mixed Methods Appraisal Tool, study data were extracted, and behaviour change techniques were coded. Recognition of online risks was taught effectively across most interventions and management of online risks had some coverage which was mostly effective. However, few interventions included the teaching of recovery from online risks, and this was rarely assessed as an outcome measure. Therefore, future interventions and curricula should aim to have a greater focus on digital resilience in its entirety in which children can recognize and manage online risks and go on to recover following exposure to online risks.

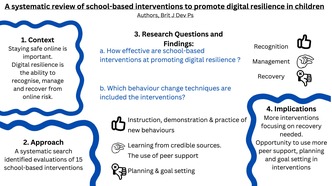

## Graphical abstract

As demonstrated through the graphical abstract, this systematic review provides insight into schoo‐based online safety interventions and the components that make these interventions effective. There was a specific focus on digital resilience and how this was identified in each intervention.

**Statement of contribution**

**What is already known on this subject?**

- Supporting children to be safe online is increasingly important.
- Digital resilience is an emerging concept which contends children also need to have the ability to recover from negative online experiences.

**What does the present study add?**

- Overall existing interventions are effective in developing children's ability to recognize and to some extent manage online risks.
- We are lacking interventions which develop children's skills to recover from negative online experiences.
- Instructions, demonstrations, and opportunities to practice are the most common behaviour change techniques used; planning and goal setting are little used.

## INTRODUCTION

The pervasiveness of digital technology—96% of UK children aged 3–17 years of age had been online in 2024 (Ofcom, 2025) while Australian children aged 12–17 years were spending on average 14.4 h per week online (eSafety Commissioner, 2021) – has led to growing concerns about how to support children to respond to online risks. Ofcom (2025) found that a third of 8–17‐year‐olds have been exposed to ‘worrying’ or ‘nasty’ content. Similarly, the eSafety Commissioner (2021) reported 44% of children surveyed had a negative experience online in 2020. Additionally, girls (34%) are somewhat more likely to be exposed to ‘worrying’ or ‘nasty’ content than boys (28%; Ofcom, 2025). However, the risks children may experience online extend beyond content and have been categorized by Livingstone and Stoilova (2021) as the 4Cs: the contact that children may experience online, the conduct children may carry out or experience online; the contracts they may be exploited by online because of digital providers and the content a child may be exposed to online. However, children commonly hold misconceptions about online risks including how their data are used and shared when they access online technologies (Starks & Reich, 2024). Similarly, although Macaulay et al. (2020) found that English children had a good awareness of the types of online risks that may occur, they were uncertain about how to keep themselves safe from these risks.

Most UK children are not discussing online risks with their parents, with only 23% of parents reporting their children talking to them about upsetting or scary content they see online (Ofcom, 2025). In contrast, Ofcom found 92% of 8–17‐year‐olds recalled having at least one lesson at school focusing on being online and the risks involved, with 45% of children stating that these lessons were useful. This report notes that the most common online safety topics children recall being taught at school were how to recognize harmful content, how to keep personal information safe, and how to be kind and respectful to others online; this is mostly about raising awareness of how to recognize online risks. However, there was little evidence of children reporting being taught how to manage or recover from online risks, which are both important aspects in the development of digital resilience skills.

The UK Council for Internet Safety's (UKCIS, 2020) Digital Resilience Framework (DRF) outlines three key components for developing children's digital resilience; (1) understand risks online, (2) know how to seek help to manage these risks, (3) learn from their experiences and recover when things go wrong online. Following publication of the DRF, Hammond et al. (2023) conducted focus groups asking children aged 8–12‐years‐old in England to discuss any occasions where they had been online and encountered risks and how they responded. This analysis resulted in digital resilience being defined as a dynamic process where individuals must learn how to recognize, *manage*, and *recover* from online risks at four levels (the self, home, community, and civil society). Management refers to children being able to navigate risks practically and emotionally, seeking support when needed (Hammond et al., 2023). In contrast, recovery from online risks refers to acceptance and sometimes (not always) growth following recognition or management of an online risk (Hammond et al., 2023). These three concepts of digital resilience inform this systematic review.

It is particularly important to ensure that children are equipped with skills to safely and effectively manage and recover from their online experiences. Otherwise experiences online may lead to negative outcomes, including challenging behaviour, sleep difficulties and depressive symptoms (Rega et al., 2023). Kokka et al. (2021) provide further support which emphasizes the negative impact of problematic internet use on children's sleep in which quality was impacted, as well as symptoms of insomnia, and Eddy Ives et al. (2025) reported associations between screen time and problematic internet use in children and adolescents with anxiety and depression, self‐injury behaviours, eating disorders and body dysmorphia, as well as ADHD and ASD.

The importance of skill development is supported by James and Prout (2015) who suggest that children's experiences during childhood are socially constructed and therefore children must be active participants who make decisions about their own experiences. Skill development, rather than information provision or awareness raising, is also more likely to help children as the types of risks children experience online vary (Livingstone & Stoilova, 2021). Furthermore, Ryff and Singer (2008) explore eudaimonic psychological well‐being in which personal growth, autonomy and purpose in life are crucial to an individual's well‐being which self‐determination theory provides support for (Deci & Ryan, 2000). These theoretical frameworks all highlight the need that humans must feel autonomous and responsible over the challenges they face in life. Therefore, it is vital that children develop the knowledge and skills to engage with technology. By developing digital resilience skills, that is, learning how to recognize, manage, and recover from online risks, children will have skill that can be used in response to a varied range of online risks.

When considering children's development of digital skills, it is important to consider where they will do this and how they will be supported in a way that is appropriate for their age and stage of development. Bronfenbrenner and Morris (2007) consider the layers where the child's environment influences their development, individually, or by inter‐layer interactions. Influences on children's digital skills occur at all levels of a child's bioecological system (Bronfenbrenner & Morris, 2007). The impact of school‐based interventions, and the policy that may influence the approach a school takes are just some of the factors that influence children's behaviour across the micro‐, meso‐ and exosystems. The Association for Child and Adolescent Mental Health (2025, October 9) explain that schools offer a good environment to support children when looking to develop their mental health and resilience, particularly early interventions implemented before a difficulty occurs. As children already receive lessons at school about being online and the risks involved (Ofcom, 2025), it is important we determine the impact such lessons have on children's awareness of risks.

Existing approaches taken by educational bodies to educate children about online safety vary globally in the extent to which they encompass the DRF components; compared with traditional teaching of online safety, the key difference the DRF provides is the idea that individuals must *engage* with online challenges and find *appropriate* responses, rather than avoiding contact with risks; UKCIS describe this as ‘digital activation’. For example, the National Curriculum in England (Department for Education, 2013, ‘Aims’ section) includes one curriculum aim relevant to the DRF: that children are taught to be ‘responsible, competent, confident and creative users of technology.’ In comparison, the Australian Curriculum (2023) has five dimensions of learning focusing on teaching children about online safety (values rights and responsibilities; well‐being; respectful relationships; digital media literacy; informed and safe use of information and devices). Similarly, the European Commission (2023) outlines that media literacy is a key focus in Belgium's curriculum which sets out clear objectives to ensure children are taught to; (1) understand the rules that exist on digital platforms, (2) evaluate the risks of behaviours online, and (3) understand the influence that the digital and non‐digital media can have on society. In addition to school curricula teacher‐led interventions have been found to increase children's awareness of risk and how to behave online (O'Higgins Norman et al., 2023; Vanderhoven et al., 2014).

In view of recently introduced definitions of digital resilience (Hammond et al., 2023; UKCIS, 2020), and due to the lack of an existing systematic review which evaluates the effectiveness of school‐based interventions against the information set out in current policy and guidance to ensure children can use digital technology in a way that enhances their use of technology while protecting their well‐being, we conducted a systematic review to clarify the extent to which pre‐existing school‐based interventions are effectively promoting the online safety skills described in the DRF. While acknowledging that these interventions did not necessarily intend to meet the purpose of teaching digital resilience as they may pre‐date the DRF, we believe there is value in mapping them onto the DRF as this is likely to serve as a guide to school‐based content in the future.

To further enhance our understanding, we used The Behaviour Change Taxonomy (BCTv1; Michie et al., 2013) to help identify if successful interventions are more likely to use certain techniques; developed by Michie et al. (2013) to allow health psychologists to identify techniques used in effective interventions, the BCT has been used across a range of disciplines including psychology and education (e.g., Al‐walah et al., 2023; Cho & Kizilcec, 2021; Watson et al., 2021) to report the content of interventions. Therefore, we used the BCT v1, which includes 16 behaviour groups with 93 individual behaviour change techniques, to code interventions included in this current review to understand *how* interventions can be effective in changing children's behaviours.

Overall, this review sought to investigate the extent to which existing school‐based interventions promoting digital safety target elements of digital resilience (recognition, management, recovery from online risks). Furthermore, to better understand the methods used in the interventions included in this review, the BCT was used to code which techniques interventions implemented. Using the BCT provides new insights into both the effectiveness of different interventions and the methods used within them. This information can be used to improve the development of future interventions. In sum, this review aimed to address two research questions:

(1) How effective are school‐based interventions at promoting digital resilience in children aged 18 or younger?

(2) Which behaviour change techniques are included in interventions which promote digital resilience in children aged 18 or younger?

## METHODS

The systematic review was pre‐registered via Prospero (CRD42023420624) and the protocol is available on the Open Science Framework (https://osf.io/yjnz2/overview?view_only=2fdf8239359f40b998a66f592133ee66). The review was carried out in accordance with the PRISMA Statement (Page et al., 2021).

### Eligibility criteria

Eligible studies reported results where participants were aged 18 years or younger and received an intervention targeting change in a measure of digital resilience using pre‐ and post‐measures of the same outcome, including mixed‐methods studies, in a school‐based setting. Digital resilience was identified using the most up‐to‐date definition of recognizing, managing and recovering from online risk (Hammond et al., 2023); two authors independently read through papers at full‐text stage to identify evidence for the presence of at least one element of digital resilience being included. Therefore, included interventions showed evidence of promoting children's digital resilience, where at least one element of digital resilience was included. Studies could be published and conducted anywhere in the world providing the report was published in English. All studies were published in peer‐reviewed journals, or were theses obtained from EThOS, and were published between 2010 and 2025. Review papers and protocol papers were excluded. Including research from 2010 onwards reflected increases in the use of social media (Our World in Data, 2019) and follows the suggestion by Aichner et al. (2021) that 2010 reflects an important time point in terms of how perceptions of social media changed from being a tool to connect people with common interests to being a platform to create, share, and access user‐generated content. While this review does not focus exclusively on social media it is recognized that this is often a key online activity that children engage in. Furthermore, our decision was based on changes in children's online behaviours reported by Ofcom at this time. Specifically, Ofcom (2009) reported that children's use of televisions and game consoles were much more common than their access to the internet in their bedroom (16% of children aged 8–11, 3% of children aged 3–7) suggesting that fewer children had direct contact with the types of online risks children are exposed to in more recent Ofcom reports (e.g., 2025). In comparison, Ofcom (2011) have a much greater focus on reporting children's uses of mobile phones, laptops, and social media services. This therefore supports the decision to focus on interventions from 2010 onwards to acknowledge the developing landscape of children's use of technology.

### Information sources

ERIC, PsycINFO, Scopus, and EThOS (for PhD theses) were initially searched in July 2024 for literature published between 2010 and 2024. A repeat search was conducted in February 2025 and October 2025, but no new studies met the inclusion criteria. Reference lists of included literature were checked for studies that might meet the inclusion criteria.

### Search strategy

The search strategy used the SPIDER approach to account for the breadth needed in the search terms for this review. This is supported by Cooke et al. (2012) who explain that SPIDER supports a more advanced searching approach which is more suitable for quantitative and qualitative research studies, as well as mixed‐methods research studies. The search terms included words and phrases relevant to the inclusion criteria regarding children and adolescents, schools and classrooms and variations of online safety and types of digital media (e.g., social media, media literacy, online risk, digital resilience) and methods (e.g., randomized control trials, quantitative, qualitative, mixed method).

### Search string

(“child*” OR “adolescen*” OR “student” OR “pupil” OR “teen*” OR “classroom” OR “school”) AND (“online safety” OR “internet” OR “social media” OR “social network*”) AND (“well being” OR “well‐being” OR “wellbeing” OR “digital resilience” OR “media literacy” OR “digital literacy” OR “online risk”) AND (“intervention” OR “program*” OR “wait‐list*” OR “control group” OR “experiment” OR “quasi” OR “randomised control trials” OR “pre comparison” OR “post comparison” OR “quantitative” OR “qualitative” OR “mixed method”).

### Selection process

Duplicates were removed in RefWorks and checked again in Rayyan. In Rayyan, the first author screened all titles and abstracts, in alphabetical order by title, to determine eligible studies. The second author screened a random 10% (*N* = 561), and there was 100% agreement for all include and exclude decisions. The first and second authors reviewed 100% of the full‐text studies. Initially, six conflicts were found when reviewing full texts; however, these were successfully resolved after re‐discussion of the details of the inclusion and exclusion criteria (specifically around the methods and outcomes of the interventions). As part of the full‐text screening, the inclusion criteria were discussed with the third author, and it was decided to include studies with a pre‐ and post‐measure, with or without a control group. Requiring a control group led to exclusion of relevant studies from this emerging literature.

Following these discussions, 13 articles were included for the systematic review from the database searches. Following this initial process, two additional articles were included for data extraction. One was included to support the already included paper by Berman and White (2013) as it reported their quantitative findings (Foundation for Young Australians, 2012). When these two articles from the same project are discussed for data extraction, BCT coding, and Quality Appraisal, they will be discussed together as they are one intervention. Sekarasih et al. (2018) were identified and included during the process of checking the reference lists of all included papers. A further article (Lee & Hancock, 2023) was found when carrying out general literature searches to facilitate the writing of the introduction. This study met all the inclusion criteria, but this had not been identified during the searches as the journal in which it was published (Computers and Education Open) was not indexed in any of the databases searched. Therefore, to make sure that no further additional relevant articles had been published in this journal, a journal specific search was carried out. All 111 titles and abstracts that were returned by the search ‘“online safety” OR “internet” OR “social media” OR “social network”’ were reviewed by the second author and no further additional articles were identified.

### Data extraction

Data extraction used the following headings: Study Characteristics; Participant Characteristics; Intervention Details; Control Group Details; Measures; Outcomes; Elements of Digital Resilience. The blank data extraction form is available on Open Science Framework (https://osf.io/yjnz2/overview?view_only=2fdf8239359f40b998a66f592133ee66). To facilitate the data extraction, Admiraal (2015) was contacted to gain more information about the scale used; this was provided in its original language (Dutch) and translated using Google Translate by the first author. The first author extracted data from all articles. To ensure the accuracy of this the second author independently extracted data from four articles, and as agreement was very good (only small differences in level of detail) subsequent extraction was reviewed and agreement reached. As part of this data extraction, each element of digital resilience (recognizing risks, managing risks, and recovering from risks) was coded from the intervention details and outcome measures and agreed by both reviewers. Behaviour Change Techniques were extracted and coded independently by the first and third authors. This involved coding each study for the presence or absence of all BCTs listed in the Behaviour Change Taxonomy v1 (Michie et al., 2013). These authors discussed results of coding for each paper and resolved any disputes by discussion.

### Study quality

The first and second authors independently quality assessed all included articles using the Mixed Methods Appraisal Tool (MMAT; 2018). Agreement was reached for all papers following discussion, except for one paper which required discussion with the third author (Vanderhoven et al., 2016a). This was due to the study reporting that they had qualitative and quantitative outcome measures; however, the qualitative outcomes were unclear. The conclusion was made that the study would be appraised as mixed methods despite the lack of clarity regarding the qualitative outcomes.

### Synthesis methods

Following data extraction, it was clear that there was heterogeneity in outcomes reported by included studies, meaning it was not possible to conduct a meta‐analysis. Therefore, a narrative synthesis (Popay et al., 2006) was conducted.

## RESULTS

**FIGURE 1:**
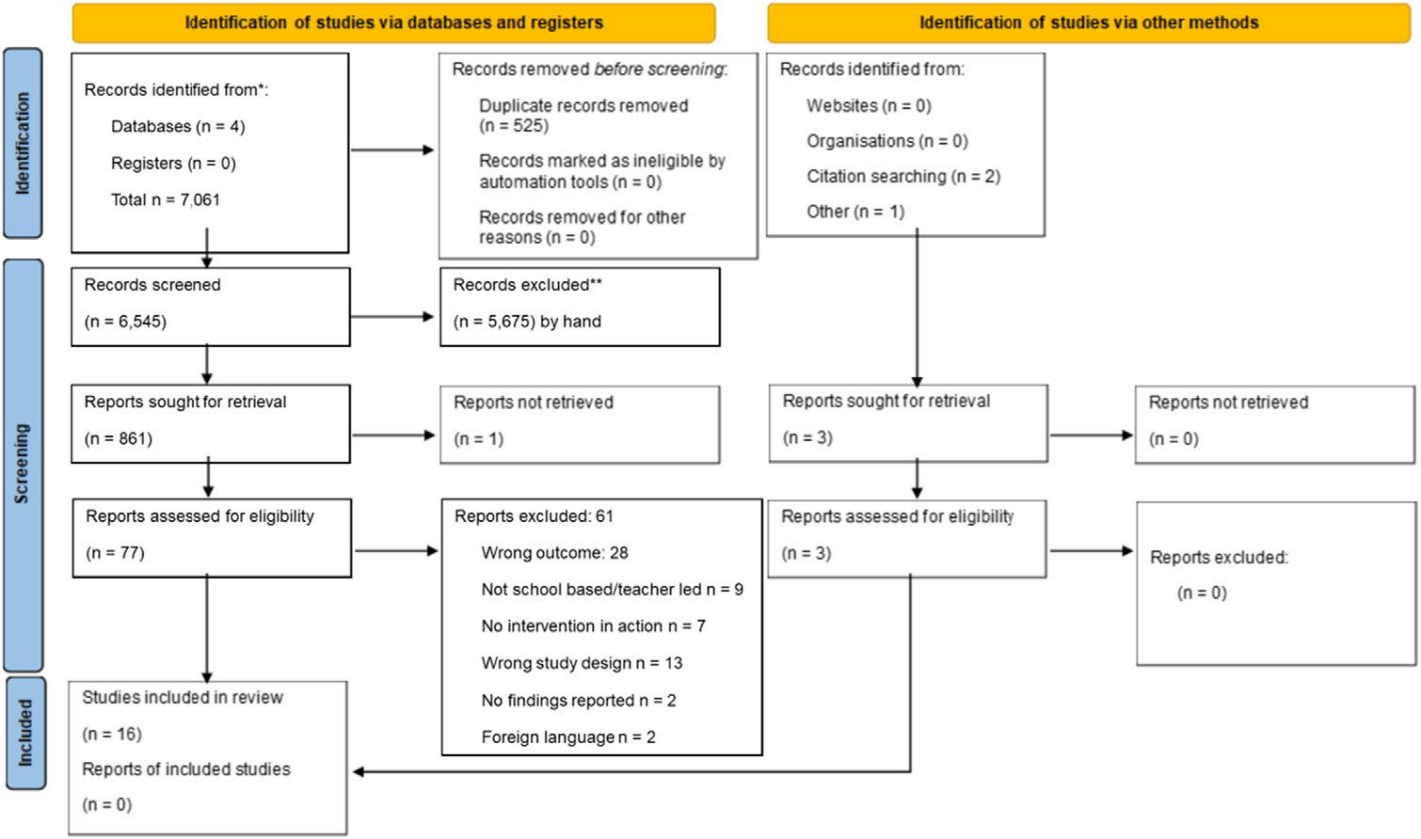
PRISMA flowchart.

Figure 1 summarizes the searching and screening processes. This resulted in 16 papers, reporting 15 interventions, identified for inclusion. Included studies' characteristics are summarized in Table 1 and further details of each study are included in Tables S1 and S2. All studies were published between 2012 and 2024, with a greater number of studies being published more recently. According to the study types defined in the MMAT (2018), four were quantitative randomized controls, nine were quantitative non‐randomized controls and two were mixed methods. Nine recruited European‐based samples, three samples were from North America, two samples were from Asia and one from Australia (ethnicity was not widely reported across the studies). Sample sizes ranged from 69 to 1399 participants (mean = 537.5, SD = 575.8, median = 306). Across studies, participants were aged 8–18 years‐old, except for Vanderhoven et al. (2014) and Vanderhoven et al. (2016b). Despite the participant age range for these studies (11–19 years) going up to 19 which falls outside the inclusion criteria of 18 years or younger, these studies were included due to the reported mean age (15.06, 14.90 years) and standard deviation (1.87, 1.88 years) of their sample indicating that almost all participating children were within the age range of focus.

**TABLE 1:** Study characteristics.

	Country of delivery	Age	Gender ratio	Sample size	Control group	Study quality
Admiraal (2015)	Netherlands	NR	30 boys, 39 girls	69	N	80%
Berman and White (2013)	Australia	13–15	15 boys, 32 girls	47	N	40%
Boulton et al. (2016)	United Kingdom	8–11	151 boys, 140 girls	291	Y	40%
Lee and Hancock (2023)	United States	Average 10–11	274 girls, 264 boys, 28 NR	566	N	80%
Liau et al. (2017)	Singapore	8–11	149 boys, 157 girls	306	Y	60%
Ondrušková and Pospíšil (2023)	Czech Republic	13–15	330 girls, 315 boys	645	N	40%
O'Rourke and Miller (2022)	Republic of Ireland	8–11	176 boys, 148 girls	324	Y	40%
Pham et al. (2024)	Vietnam	13–15	NR	1399	Y	60%
Schilder et al. (2016)	Belgium	8–14	399 boys, 420 girls	819	Y	0
Sekarasih et al. (2018)	United States	Mean: 10.53	50.4% boys, 49.6% girls	117	N	60%
Theophilou et al. (2023)	Spain	Mean: 14.7	52% boys, 47.9% girls	142	N	60%
Vanderhoven et al. (2014)	Belgium	11–19	NR	2071	Y	60%
Vanderhoven et al. (2016a)	Belgium	12–14	36% boys, 64% girls	203	Y	60%
Vanderhoven et al. (2016b)	Belgium	11–19	NR	974	Y	60%
Wascher (2021)	United Stated	8–11	41 boys, 54 girls, 1 non‐binary	89	N	80%

Abbreviation: NR, not reported.

### Quality analysis

The studies were rated based on the MMAT (2018) criteria for quality and a percentage was calculated to score the overall quality (reported in Table 1). Overall, the quality was variable with three studies scoring 80%, seven scoring 60%, four scoring 40%, and one scoring 0% quality. The full‐quality assessment is available in Table S3. To aid the reader in considering the quality of the studies as we analyse their findings within this narrative synthesis, we will use a scale staring system each time we refer to a study, with 80% quality being represented by **** and 20% by * and the study with a quality score of 0 receiving no stars.

### RQ1: How effective are school‐based interventions at promoting digital resilience in children aged 18 or younger?

The three elements of digital resilience defined by Hammond et al. (2023) were used to discuss the outcomes of the included interventions; Table 2 displays where these elements were targeted in the intervention and/or assessed in the outcome measure(s) of each study. While all interventions aimed to improve children's ability to recognize risk, some aimed to improve management skills, and most assessed these as outcomes, only two interventions aimed to improve recovery from risk, and two (but not the same two) assessed this as an outcome measure.

**TABLE 2:** Digital resilience components.

Author		Admiraal (2015)	Berman and White (2012)	Boulton et al. (2016)	Lee and Hancock (2023)	Liau et al. (2017)	Ondrušková and Pospíšil (2023)	O'Rourke and Miller (2022)	Pham et al. (2024)	Schilder et al. (2016)	Sekarasih et al. (2018)	Theophilou et al. (2023)	Vanderhoven et al. (2014)	Vanderhoven et al. (2016a)	Vanderhoven et al. (2016b)	Wascher (2021)
Element of digital resilience																
Intervention	Recognition	✔	✔	✔	✔	✔	✔	✔	✔	✔	✔	✔	✔	✔	✔	✔
	Management	?	?	?	✔	✔	✔	?	✔	?	✔	✔	✔	?	?	✔
	Recovery	X	X	X	✔	X	X	X	X	?	X	✔	X	X	X	X
Outcome measures	Recognition	X	✔	✔	✔	✔	✔	X	✔	✔	✔	✔	✔	✔	✔	✔
	Management	✔	X	✔	✔	X	✔	X	✔	✔	X	✔	✔	✔	✔	✔
	Recovery	X	X	X	✔	X	?	✔	?	X	X	?	X	X	X	X

*Note*: ✔, included;?, unclear; X, not included.

#### Recognizing online risks

Thirteen studies included outcome measures related to recognizing online risks (Berman & White, 2012**; Boulton et al., 2016**; Lee & Hancock, 2023****; Liau et al., 2017***; Ondrušková & Pospíšil, 2023**; Pham et al., 2024***; Schilder et al., 2016; Sekarasih et al., 2018***; Theophilou et al., 2024***; Vanderhoven et al., 2014, 2016a, 2016b***; Wascher, 2021****). Recognizing risks was assessed via children's awareness, attitudes, and knowledge of online risks.

Six interventions measured awareness or attitudes towards risk of harm young people may face when interacting with other users online, with two of these also considering risks associated with inappropriate content. Three interventions (Vanderhoven et al., 2014, 2016a, 2016b***) were successful in increasing children's self‐reported awareness of ‘contact risks’ but only Vanderhoven et al. (2016b)*** found evidence for changing attitudes towards contact online risk and only in the intervention which involved individual reflection. In addition to contact risks Vanderhoven et al. (2014)*** considered content and commercial risks, again self‐reported awareness of these increased after the intervention, but attitudes towards these risks remained unchanged. Correspondingly, Liau et al. (2017)*** found that after the intervention mentees reported significant changes in attitudes towards meeting someone in person that they had initially met online, making online disclosures and cyberbullying, but that attitudes towards inappropriate content did not alter. Similarly, Boulton et al. (2016)** reported an intervention involving older peers teaching younger peers and found self‐reported risk awareness only increased for the peer tutors, not the tutees. Finally, Schilder et al. (2016) found using self‐report measures that both directly after their intervention, and at a 4‐month follow up, those who experienced the intervention had greater awareness of the risks of interacting with others online compared with those in the control group. Therefore, all these interventions were found to improve children's awareness of risks they may face when interacting with others online to some extent. However, not all aspects of all interventions were successful, findings relied on self‐reports and the only study to consider longer term retention of risk awareness (Schilder et al., 2016) was of low quality.

Five interventions measured children's awareness and knowledge of digital privacy and cybersecurity. Lee and Hancock (2023)**** found that following the intervention children reported greater awareness of digital privacy issues. Similarly, Pham et al.'s (2024)*** intervention group showed greater awareness of the risks of sharing personal information online compared with their control group. Correspondingly, Ondrušková and Pospíšil (2023)** discussed an increase in cybersecurity awareness assessed using scenarios following the intervention suggesting that children could better recognize online risks in relation to cybersecurity. No further specific conclusions could be identified. However, their findings are supported by Theophilou et al. (2023)*** who found that intervention children's reflections on their self‐protection skills when using social media increased. Lastly, Wascher (2021)**** found that children's self‐reported knowledge of media literacy concepts, including personal safety improved after the intervention. Therefore, all five interventions demonstrated positive outcomes in terms of increasing children's awareness or knowledge of the importance of digital privacy and these studies were generally of good quality, although most relied on self‐report measures.

Two interventions measured children's ability in recognizing implicit online risks that might be embedded in the media they consume, specifically, perceptions of gender in the media (Berman & White, 2013**) and hidden messages in advertising (Sekarasih et al., 2018***). While one study (Berman & White, 2013**) found a range of positive outcomes, the other (Sekarasih et al., 2018***) found some positive outcomes, but also no change in some other outcomes. Therefore, although most interventions were effective in increasing children's ability to recognize risks, some outcomes did not change following interventions.

#### Managing online risks

Eleven interventions measured children's ability to manage online risks (Admiraal, 2015****; Boulton et al., 2016**; Lee & Hancock, 2023****; Ondrušková & Pospíšil, 2023**; Pham et al., 2024****; Schilder et al., 2016; Theophilou et al., 2024***; Vanderhoven et al., 2014, 2016a, 2016b***; Wascher, 2021****). These outcomes related to children's behaviours when managing their own online presence, interacting with others online, and managing risks associated with content available online.

Five studies assessed children's abilities to take steps to manage their online presence. Vanderhoven et al. (2016a)*** found that pupils reported less intention to post‐personal information online and 13% reported making changes to increase their online safety. Similarly, Vanderhoven et al. (2016b)*** found that those who received the interventions were more likely to report making changes to their online profiles (like altering privacy settings) compared with those in the control group. Relatedly, Theophilou et al. (2023)*** found that an intervention was successful in improving children's self‐reported knowledge of how to manage their social media account, including what content to share, adjusting privacy settings, and unfollowing social media users who are negatively influencing their mood. Correspondingly, Pham et al. (2024)*** found increased online account security practices and reduced identity disclosure on social networking sites among intervention compared with control group participants at the post‐test. Furthermore, Boulton et al. (2016)** found that both peer tutors and tutees reported a significantly improved knowledge of safety including avoiding accidentally sharing personal information and photographs after the peer‐delivered intervention. Therefore, all these interventions show evidence of improving children's ability to manage their personal information online.

Seven interventions assessed children's ability to manage online risks in relation to interacting with others online. Pham et al. (2024)**** found that children who experienced an online safety intervention, compared with a control group, were less likely to report accessing links sent by strangers and accept friend requests from people they had not met in person. Similarly, using an online task with interactive scenario‐based activities, Ondrušková and Pospíšil (2023)** found that after the intervention children's ability to resist messages containing harassing materials and asking for contact information had increased. Furthermore, Lee and Hancock (2023)**** found that following an intervention, children reported higher self‐efficacy regarding standing up for themselves online. However, following a peer‐delivered intervention Boulton et al. (2016)**, found that peer tutors, but ot peer‐tutees, reported a significantly improved knowledge of how to manage cyberbullying. Mixed results were also found by Vanderhoven et al. (2016b)*** as they reported that an intervention involving individual reflection, but not one involving collaborative learning, had a positive impact on children's behaviours relating to contact risks. Similarly, Vanderhoven et al. (2014)*** reported mixed results as they found that an intervention focusing on contact risks had no direct effect on behaviours for managing contact risks, but an intervention designed to increase awareness of content risks did increase behaviours related to managing contact risks, the authors suggested this was due to overlap in the course content. Finally, Schilder et al.'s (2016) intervention had no significant impact in reducing risky behaviours related to interacting with others online, however it should be noted that this study was of low methodological quality. Nonetheless, it seems that interventions have mixed effectiveness in developing children's abilities of how to manage interacting with others online effectively across studies of varying quality.

Three interventions assessed children's ability to manage risks associated with content they might encounter online. Boulton et al. (2016)** found that a peer facilitated intervention was successful in increasing both tutors and tutees self‐reported knowledge of how to avoid a computer virus. Relatedly, Admiraal (2015)**** measured children's reflective internet skills for uploading and downloading information online. Girls were found to outperform boys' skills for safely uploading and downloading information at the pre and post‐tests, however, boys reported significant increases in their skills following the intervention, whereas girls' scores were maintained. One further intervention (Wascher, 2021****) which involved assessing children's self‐reported skills in managing a wide range of potential online risks, including asking a trusted adult before sharing personal information online, telling a trusted adult when they are exposed to scary or hurtful content upsetting content and whether they know what to do when they see cyberbullying also reported that scores on the self‐report measure increased from pre‐ to post‐test. Therefore, these three interventions all reported positive outcomes, but all relied on self‐report data.

#### Recovering from online risks

Only two interventions measured recovering from online risks. O'Rourke and Miller (2022)** measured children's recovery from online risks by measuring their well‐being 10 weeks post‐intervention and found that self‐reported post‐test well‐being was significantly higher in the intervention group compared with the control group. Lee and Hancock's (2023)**** measures included upstander intentions online where students were asked to reflect on their ability to support others who were being bullied online and help‐seeking behaviours in which students were asked to reflect on their willingness to seek help from trusted caregivers and peers. They found that students' upstander efficacy was higher after the intervention, with girls' intentions significantly improving following the intervention. Students were also more likely to seek support for difficult online situations following the intervention, with significance found when seeking help from trusted adults (specifically parents and teachers). Therefore, both interventions which looked at recovery reported positive outcomes.

### RQ2: Which behaviour change techniques are included in interventions which promote digital resilience in children aged 18 or younger?

Eighteen distinct behaviour change techniques were coded from ten of the behaviour change groups within the Behaviour Change Taxonomy (Michie et al., 2013) across the 15 interventions included for analysis (Table 3). Fourteen of these techniques demonstrated successful outcome measures in relation to at least one digital resilience component. The most commonly reported BCTs were behavioural practice/rehearsal (8.1, *n* = 12), instruction (4.1, *n* = 11), and demonstration (6.1, *n* = 11). Behavioural practice/rehearsal included tasks where children responded to online safety scenarios, like not having a strong password. Instruction to perform the behaviour included video, written, or verbal instructions. Demonstration of the behaviour included showing children how to make a strong password, role‐playing online safety scenarios, and guided activities within digital platforms.

**TABLE 3:** Behaviour change techniques.

	1.2	2.2	3.1	3.2	3.3	4.1	5.1	5.3	5.6	6.1	6.2	6.3	8.1	9.1	9.2	11.2	12.3	15.3
Admiraal (2015)	***✔***												***✔***		***✔***			
Berman and White (2013)														***✔***				
Boulton et al. (2016)				***✔***		***✔***		***✔***	***✔***	***✔***			***✔***	***✔***		***✔***	***✔***	
Lee and Hancock (2023)			***✔***			***✔***		***✔***		***✔***			***✔***	***✔***				***✔***
Liau et al. (2017)		***✔***	***✔***			***✔***				***✔***			***✔***	***✔***				
Ondrušková and Pospíšil (2023)						***✔***		***✔***		***✔***			***✔***					
O'Rourke and Miller (2022)				***✔***		***✔***				***✔***			***✔***	***✔***				
Pham et al. (2024)			***✔***			***✔***				***✔***			***✔***					
Schilder et al. (2016)								***✔***						***✔***				
Sekarasih et al. (2018)							***✔***						***✔***	***✔***				
Theophilou et al. (2023)						***✔***				***✔***			***✔***					
Vanderhoven et al. (2014)				***✔***		***✔***				***✔***			***✔***	***✔***				
Vanderhoven et al. (2016a)				***✔***	***✔***	***✔***				***✔***			***✔***	***✔***				
Vanderhoven et al. (2016b)			***✔***			***✔***				***✔***	***✔***	***✔***	***✔***					
Wascher (2021)						***✔***				***✔***			***✔***	***✔***				

Credible sources were frequently used (9.1, *n* = 9) and included researchers, materials from websites such as Common‐Sense Media as well as peer mentors and class teachers. Social support was also frequently used (3.1 unspecified *n* = 4, 3.2 practical *n* = 4, 3.3 emotional *n* = 1). This included older children teaching younger year groups and the use of role‐play activities. Other codes (reduce negative emotions (11.2, *n* = 1) and avoidance/reducing exposure to cues for the behaviour (12.3, *n* = 1)) were less frequently coded; this may reflect the lack of emotional or sensitive material discussed during the interventions. In contrast, techniques which related to consequences of behaviour were used five times (5.3 information about social and environmental consequences, *n* = 4 and 5.6 information about emotional consequences, *n* = 1).

All other techniques that were coded appeared only once across the sample of interventions (1.2 problem‐solving, 2.2 feedback on behaviour, 6.2 social comparison, 6.3 information about others' approval and 9.2 pros and cons, 15.3 focus on past successes). One intervention did not demonstrate a successful digital resilience outcome. In this intervention (Sekarasih et al., 2018***), children's recognition of risk was not improved despite the presence of three behaviour change techniques. Two of these techniques were seen in other interventions that had success in their outcomes (8.1 and 9.1) and one technique was unique to this intervention (BCT 5.1 information about health consequences).

## DISCUSSION

The aim of this systematic review was to answer two questions: how effective are school‐based interventions at promoting digital resilience in children aged 18 or younger and which behaviour change techniques are included in interventions which promote digital resilience in children aged 18 or younger? The 15 interventions identified for inclusion were of variable quality, included a limited range of BCTs to develop children's digital resilience and while children's skills to recognize, and to some extent manage risks online, increased following the interventions, few interventions improved recovery from online risks, and this was rarely included in the content or assessed as an outcome measure. The structure of this discussion has followed the PRISMA Checklist requirements (Page et al., 2021) to provide a general interpretation of the results, discuss any limitations of the evidence included and review processes used, and the implications for practice, policy and future research.

### Overall completeness and applicability of evidence

Considering the three elements of digital resilience, 13 interventions showed some evidence of improving children's skills in recognizing online risks, 10 interventions showed some evidence of improving children's abilities to manage online risks, and two interventions showed evidence of developing children's abilities to recover from online risks. Only one intervention, which aimed to increase children's ability to manage risk, was not found to be successful at all; all other interventions had at least some success for specific groups or specific outcome measures.

Children are being taught to recognize risk in most of the interventions explored, with improvements reported in their knowledge of the risks of interacting with others and risks relating to digital privacy. Fewer interventions focused on the risks relating to inappropriate content, and the success of these interventions was more variable. Interventions aiming to develop children's skills in managing their online presence were found to be relatively successful. However, interventions to improve their behaviour when interacting with others are found to be less consistently effective.

Recovery from online risks was the least prevalent element of digital resilience covered and assessed in the interventions, suggesting that this area lacks focus within school‐based interventions. There was little guidance included in the interventions on what children can do to learn from and recover from risks they may have encountered online. This has significant developmental implications as Holloway et al. (2013) explain that young children are less resilient and therefore become more distressed when they have negative experiences online. Without providing children with the skills to be resilient following a negative experience, it is possible that this may have consequences for their later development regarding recovery from negative or emotional situations.

Of the Behaviour Change Techniques identified in the interventions, elements that ensure the teaching and learning of new knowledge were the most identified, specifically including instructions, demonstrations, and practice opportunities for new behaviours and learning from credible sources. Techniques related to peer support were seen less frequently across the interventions. However, when peer support was used it was clear that it was important to the success of the intervention, with many children reporting that they learned lots from their peers; this was particularly evident in Boulton et al. (2016)** and Liau et al. (2017)*** mixed‐methods study. The use of peer‐to‐peer support can be underpinned by key developmental theories such as Vygotsky and Bronfenbrenner's BioEcological Systems Theory (Bronfenbrenner & Morris, 2007) and supported by Baria and Gomez (2022) finding that social support can lead to positive student learning and development, suggesting that peer‐to‐peer learning can be a positive approach to children developing effective digital skills.

Few interventions used techniques associated with goal setting and planning, with just one technique from this group being coded (problem‐solving). This was surprising as goal setting is frequently used in schools to enable children to take charge of their own learning and decide what they want to achieve (Sheeran et al., 2024). Similarly, techniques such as giving feedback and receiving information regarding the consequences for behaviours were infrequently present in these interventions. Like goal setting, the use of feedback to further children's learning has been found to be important in schools (Brooks et al., 2021; Ni, 2025), and it is therefore surprising this was not included in more interventions. One possible reason for this is that many of the interventions have been developed by researchers, rather than those working in schools, and therefore they may be less familiar with the teaching approaches most used in classroom settings.

This review also questions whether an intervention will always be successful when BCTs are included. Sekarasih et al. (2018) demonstrated that while children's skills for recognizing risks were being measured, the findings did not show successful behaviour change for the children's recognition skills despite three techniques being present in the intervention. Therefore, as the BCTs present in this intervention were also included in successful interventions evaluated in this review, it cannot be said that the BCTs used can individually make an intervention successful. Another important finding from this review is that while current interventions are generally successful at increasing recognition of risk and management of risk, they are not drawing on BCTs known to be effective at changing behaviour, such as goal setting and feedback. Future interventions should be developed to test these effective BCTs in the context of digital resilience, given their success in other contexts.

### Limitations of the evidence included in the review

Research quality is variable among the included papers, as indicated by the range of quality scores on the MMAT. Furthermore, across the studies there were few established scales used. Many articles used questionnaires created for the purpose of the intervention or a combination of pre‐existing scales that were amended for the purpose of the research.

As already outlined, a wide range of measures were used to measure related online safety outcomes. This proposed several difficulties when trying to synthesize these interventions. Excluding Lee and Hancock (2023), none of the interventions explicitly aimed to target or measure digital resilience and therefore while the synthesis makes suggestions about how children's digital resilience was supported through these interventions, these are suggestions made in the context of Hammond et al.'s (2023) definition of digital resilience and not related to the aims of the researchers for the interventions included and therefore it cannot be stated that digital resilience definitely did or did not improve as a result of the interventions. Furthermore, this review and the general literature searches to contextualize this review revealed that validated measures of digital resilience are currently scarce. The variation across the studies for the scales used helps to form an argument that to efficiently evaluate the effectiveness of these interventions and how well they improve children's knowledge and/or skills, there needs to be more consistency in the measures used.

It is possible that future research may benefit from considering different ways of measuring children's digital resilience skills through completion of scenario tasks, rather than self‐report scales. Several of the studies discussed had elements that involved children responding to an online situation through a scenario such as identifying privacy risks in a fictitious social media profile (Vanderhoven et al., 2016a) or having their behaviour monitored on a purpose‐built social media platform (Theophilou et al., 2023). It is also important to consider how these skills can be measured with different age groups across different school curricula and countries. These measures must be meaningful for the children, but also, ideally, allow for comparison across research studies.

It is also challenging to suggest that behaviour changes took place because of each intervention as the outcome measures often focused on outcomes not related to BCTs that were present in the interventions. Therefore, a behaviour change technique may be present in a successful intervention; however, the success of the intervention may not be evidence of behaviour change. Furthermore, in this sample of studies, it was uncommon that findings were followed up at a later point following the initial post‐test measure; therefore, it is not possible to say that any effects seen were maintained.

While it is acknowledged that there are age and gender differences in children's use of technology and experiences on technology (Ofcom, 2025), the studies included in this review lacked detail in the reporting of many demographic characteristics including age and gender, as well as ethnicity meaning that likely age and gender differences cannot be evaluated using their findings. Future research would benefit from providing greater levels of reporting about the characteristics of their participants as well as considering these characteristics when carrying out analysis of data when considering children's knowledge and experiences when using digital technology.

### Potential biases in the review process

As demonstrated through the identification of Lee and Hancock's (2023) article, there are limitations to systematic searching. It is possible to miss papers if an article is not available on the databases used. Furthermore, the influence of publication bias on this review must also be acknowledged, as it is more likely that interventions found to be successful will be published rather than those not found to be successful. Considering these non‐significant findings would be important to gain a fuller understanding of the extent to which school‐based interventions have the potential to be effective in increasing children's digital resilience and which BCTs are found in successful compared with unsuccessful interventions. While the BCT approach is not commonly applied outside of health, it could be argued that the application of this in the current study is a strength as it has allowed for the systematic appraisal of the techniques used within the interventions.

Two interventions were excluded from the synthesis as one included no pre‐test measure and the other included no pre/post‐test; instead, they included a comparison of the control and experimental group after the intervention, and so the effectiveness of the interventions could not be confirmed (O'Higgins Norman et al., 2023; Shyshak et al., 2024). Both studies delivered interventions which included elements related to recognizing and managing online risks, and results suggested that both interventions had success for students who received the intervention. The exclusion of these two papers based on methodological criteria suggests that while research in this area is being conducted, the quality is variable. This is further reflected by our reconsideration of our initial decision to only include studies where a control as well as intervention group was included. We chose to include the more methodologically robust research within this area so that we could be confident in the conclusions that we drew; however, it must be acknowledged that this may have resulted in other potentially promising interventions not being included as they have not been robustly evaluated.

### Implications for practice and future research

The results of this review suggest that existing interventions can increase children's digital resilience in several ways. First, more learning opportunities which cover management of risk and recovery from negative experiences are required for children to develop their digital resilience skills. Second, we found that two BCTs consistently associated with successful outcomes in other fields, goal setting (Sheeran et al. 2024) and feedback (Park et al., 2014), are not being used to inform digital resilience interventions. We see this as a missed opportunity, as these are learning techniques which children will be familiar with. Future interventions testing the effects of these two BCTs on digital resilience would make a useful contribution to the literature. Third, we recommend future interventions include peer support; nine interventions in this review included this BCT, and they all showed a positive impact on digital resilience skills. Finally, considering the relationship schools develop with parents of their children, future interventions should aim to encourage schools and parents to work together across a child's mesosystem to ensure digital resilience skills developed in school can be practiced in the home, an approach found to be important when considering how children learn (Stanley & Kuo, 2022).

New research aiming to evaluate interventions needs to be rigorously designed; the quality of the studies included within this review was variable. Future evaluations should consider measures of effectiveness that relate to the digital resilience components specifically, that is, how does an intervention promote recognition/management/recovery? Research in this area would also benefit from development of a validated scale, or scenario‐based tasks, to accurately measure digital resilience skills to ensure that interventions are being reliably tested for their effectiveness in improving children's skills. Evaluations should also consider participant characteristics (e.g., gender, age) to provide a more nuanced evaluation of intervention effectiveness.

## CONCLUSION

This review provides evidence that existing school‐based interventions designed to increase children's safety online show promise – results largely suggest that these interventions successfully increase children's ability to recognize and manage online risks. However, few existing interventions focus on increasing children's ability to recover from online risks. Therefore, more attention needs to be given to interventions which address learning and recovering from negative online experiences.

## AUTHOR CONTRIBUTIONS

**Georgina C. Halliwell:** Conceptualization; investigation; data curation; project administration; methodology; formal analysis; writing – original draft; writing – review and editing. **Sarah E. Rose:** Conceptualization; methodology; formal analysis; supervision; writing – review and editing; writing – original draft. **Richard Cooke:** Conceptualization; methodology; formal analysis; writing – review and editing; supervision. **Daniel Herron:** Conceptualization; methodology; supervision; writing – review and editing.

## CONFLICT OF INTEREST STATEMENT

There are no conflicts of interest.

## Supporting information


Table S1.

## Data Availability

The data that support the findings of this study are available in the Tables S1–S3 of this article (Templates available at: https://authorservices.wiley.com/author‐resources/Journal‐Authors/open‐access/data‐sharing‐citation/data‐sharing‐policy.html).
