# Supplementary material for: A systematic review of school‐based interventions to promote digital resilience in children
Source: Br J Dev Psychol. 2026 Feb 18;44(3):621–40. doi: 10.1111/bjdp.70038 (PMC13452001; doi:10.1111/bjdp.70038)
Supplement: Supplementary file 1 [file BJDP-44-621-s001.docx]

| Table 1 | | | | | | | | |
| --- | --- | --- | --- | --- | --- | --- | --- | --- |
|  | Country of Delivery | Age | Gender Ratio | Ethnicity | School Years | Sample Size | Control Group | Study Quality |
| *Admiraal (2015)* | Netherlands | NR | 30 boys, 39 girls | NR | Grade 7 | 69 | N | 80% |
| *Berman & White (2012)* | Australia | 13-15 | 15 boys, 32 girls | Australian: 39  Other: 8 | Year 8 | 47 | N | 40% |
| *Boulton et al. (2016)* | United Kingdom | 8-11 | 151 boys, 140 girls | NR | Year 3, Year 4 | 291 | Y | 40% |
| *Lee & Hancock (2023)* | United States | Average 10-11 | 274 girls, 264 boys, 28 NR | 65% BIPOC   35% white | Fifth Grade | 566 | N | 80% |
| *Liau et al. (2017)* | Singapore | 8-11 | 149 boys, 157 girls | 48.9% Chinese   24.5% Malay   11% Indian   15.6% other | Grades 4, 5 & 6 | 306 | Y | 60% |
| *Ondrušková & Pospíšil (2023)* | Czech Republic | 13-15 | 330 girls, 315 boys | NR | Elementary school | 645 | N | 40% |
| *O'Rourke & Miller (2022)* | Republic of Ireland | 8-11 | 176 boys, 148 girls | NR | Year 5, Year 6 | 324 | Y | 40% |
| *Pham et al. (2024)* | Vietnam | 13-15 | NR | Kinh Muong | Grades 7, 8 & 9 | 1399 | Y | 60% |
| *Schilder et al. (2016)* | Belgium | 8-14 | 399 boys, 420 girls | NR | Fourth & Fifth Grade | 819 | Y | 0 |
| *Sekarasih et al. (2018)* | United States | Mean: 10.53 | 50.4% boys, 49.6% girls | NR | Fourth & Eighth Grade | 117 | N | 60% |
| *Theophilou et al. (2024)* | Spain | Mean: 14.7 | 52% boys, 47.9% girls | NR | High School | 142 | N | 60% |
| *Vanderhoven et al. (2014)* | Belgium | 11-19 | NR | NR | NR | 2071 | Y | 60% |
| *Vanderhoven et al. (2016a)* | Belgium | 12-14 | 36% boys, 64% girls | NR | First 3 years of secondary school | 203 | Y | 60% |
| *Vanderhoven et al. (2016b)* | Belgium | 11-19 | NR | NR | Unknown secondary classes | 974 | Y | 60% |
| *Wascher (2021)* | United Stated | 8-11 | 41 boys,   54 girls,   1 non-binary | 87.5% (white)   6.3% (mixed)   2.1% (black)   2.1% (native)   1% (Hispanic/ Latino/other) | Fourth & Fifth Grade | 89 | N | 80% |
|  | | | | | | | | |

| Table 2  Intervention Details | | | | | | |
| --- | --- | --- | --- | --- | --- | --- |
| Study | Theory | Facilitator | Focus | Location | Method | Lesson/Frequency |
| *Admiraal (2015)* | None | Teacher | Promoting a fictional celebrity | School | Digital role-play game | 12-18 |
| *Berman & White (2012)* | None | Teacher | Body image in adverts | School | Peer-to-peer teaching | 6 modules |
| *Boulton et al. (2016)* | Role Theory, Cognitive Theory, Vygotsky's sociocultural theory | Teacher | Knowledge of online risks and safety | School | Peer-to-peer teaching | 4, 60-minute sessions |
| *Lee & Hancock (2023)* | Bronfenbrenner’s Social ecological theory, Bandura self-efficacy, Bystander Intervention Model | Pair of trained educated | Digital life and wellbeing | School | Lectures and discussions | 1 |
| *Liau et al. (2017)* | Constructivist learning, Vygotsky's socio-constructivist theory, Theory of Planned Behaviour (TPB) | Teacher | Tackling cyber risks | School/ iZ Hero Museum exhibition | Peer-to-peer teaching | N/A |
| *Ondrušková & Pospíšil (2023)* | None | Lecturer | Cyber security | School | Lessons | 2 |
| *O'Rourke & Miller (2022)* | Subjective wellbeing, Message Interpretation Process | Teacher | Advertising and media | School | Lessons and activities | 4 lessons, 8 activities |
| *Pham et al. (2024)* | None | Researcher | Risk recognition and protective strategies | School | Training in classroom and social media platforms | 1 3-hour lesson & follow up tasks |
| *Schilder et al. (2016)* | Classification of categories of risk, TPB, Behaviour Change Model | Researcher | Risk areas on the internet | School | 10-minute presentation | 1 |

| *Table 2 Continued*  Intervention Details | | | | | | |
| --- | --- | --- | --- | --- | --- | --- |
| Study | Theory | Facilitator | Focus | Location | Method | Lesson/Frequency |
| *Sekarasih et al. (2018)* | None | Researcher | Messages in advertising | School | Lessons, activities, homework tasks and discussions | 4, 1-hour visits |
| *Theophilou et al. (2024)* | None | Unclear | Hidden risks on social media | School | Instareal, purpose-built platform | 6, 90-minute sessions |
| *Vanderhoven et al. (2014)* | Classification of categories of risk,  Transtheoretical model of Behaviour Change | Teacher | Contact, content and commercial risks | School | Peer tasks, discussions, theory | 1 hour, 1 per risk type |
| *Vanderhoven et al. (2016a)* | Classification of categories of risk, TPB | Teacher | Contact risks | School | Homework task, then intervention | 1 hour |
| *Vanderhoven et al. (2016b)* | TPB, Classification of categories of risk,  Transtheoretical model of Behaviour Change | Teacher | Social network site profile | School | Collaborative and reflective learning | 1 per condition |
| *Wascher (2021)* | Styles of parenting | Researcher | Media literacy skills | School | 3 videos followed by discussions | 2, pre-test and intervention, post-test |

| *Table 3*  *Full MMAT reporting* | | | | | | | | | | | | | | | | | | | | | | | | | | |
| --- | --- | --- | --- | --- | --- | --- | --- | --- | --- | --- | --- | --- | --- | --- | --- | --- | --- | --- | --- | --- | --- | --- | --- | --- | --- | --- |
| Studies | Criteria from the Mixed Methods Appraisal Tool | | | | | | | | | | | | | | | | | | | | | | | | | |
|  | 1.1 | 1.2 | 1.3 | 1.4 | 1.5 | 2.1 | 2.2 | 2.3 | 2.4 | 2.5 | 3.1 | 3.2 | 3.3 | 3.4 | 3.5 | 4.1 | 4.2 | 4.3 | 4.4 | 4.5 | 5.1 | 5.2 | 5.3 | 5.4 | 5.5 |  |
| Admiraal. 2015 |  |  |  |  |  |  |  |  |  |  | 0 | 1 | 1 | 1 | 1 |  |  |  |  |  |  |  |  |  |  |  |
| Berman & White 2013 | 1 | 1 | 0 | 0 | 0 |  |  |  |  |  | 0 | 1 | 0 | 1 | 1 |  |  |  |  |  | 1 | 1 | 1 | 1 | 0 |  |
| Boulton et al. 2016 |  |  |  |  |  | 0 | 0 | 1 | 0 | 1 |  |  |  |  |  |  |  |  |  |  |  |  |  |  |  |  |
| Lee & Hancock. 2023 |  |  |  |  |  |  |  |  |  |  | 0 | 1 | 1 | 1 | 1 |  |  |  |  |  |  |  |  |  |  |  |
| Liau et al.  2017 |  |  |  |  |  |  |  |  |  |  | 0 | 1 | 0 | 1 | 1 |  |  |  |  |  |  |  |  |  |  |  |
| Ondrušková & Pospíšil. 2023 |  |  |  |  |  |  |  |  |  |  | 0 | 1 | 1 | 0 | 0 |  |  |  |  |  |  |  |  |  |  |  |
| O'Rourke & Miller. 2022 |  |  |  |  |  | 1 | 1 | 0 | 0 | 0 |  |  |  |  |  |  |  |  |  |  |  |  |  |  |  |  |
| Pham et al. 2024 |  |  |  |  |  | 1 | 1 | 1 | 0 | 0 |  |  |  |  |  |  |  |  |  |  |  |  |  |  |  |  |
| Schilder et al. 2016 |  |  |  |  |  | 0 | 0 | 0 | 0 | 0 |  |  |  |  |  |  |  |  |  |  |  |  |  |  |  |  |
| Sekarasih et al. 2018 |  |  |  |  |  |  |  |  |  |  | 0 | 1 | 0 | 1 | 1 |  |  |  |  |  |  |  |  |  |  |  |
| Theophilou et al. 2024 |  |  |  |  |  |  |  |  |  |  | 0 | 1 | 0 | 1 | 1 |  |  |  |  |  |  |  |  |  |  |  |
| Vanderhoven et al. 2014 |  |  |  |  |  |  |  |  |  |  | 0 | 1 | 0 | 1 | 1 |  |  |  |  |  |  |  |  |  |  |  |
| Vanderhoven et al. 2016(a) | 1 | 1 | 1 | 1 | 1 | 0 | 1 | 1 | 0 | 1 |  |  |  |  |  | 1 | 1 | 1 | 0 | 1 | 1 | 1 | 1 | 1 | 0 |  |
| Vanderhoven et al 2016(b) |  |  |  |  |  |  |  |  |  |  | 0 | 1 | 0 | 1 | 1 |  |  |  |  |  |  |  |  |  |  |  |
| Wascher 2021 |  |  |  |  |  |  |  |  |  |  | 0 | 1 | 1 | 1 | 1 |  |  |  |  |  |  |  |  |  |  |  |
| *0 is coded when quality criteria is not met, 1 is coded when quality criteria is met.* | | | | | | | | | | | | | | | | | | | | | | | | | | |

| Studies | Admiraal. 2015 | Berman & White 2013 | Boulton et al. 2016 | Lee & Hancock. 2023 | Liau et al. 2017 | Ondrušková & Pospíšil. 2023 | O'Rourke & Miller. 2022 | Pham et al. 2024 | Schilder et al. 2016 | Sekarasih et al. 2018 | Theophilou et al. 2024 | Vanderhoven et al. 2014 | Vanderhoven et al. 2016(a) | Vanderhoven et al. 2016(b) | Wascher. 2021 |
| --- | --- | --- | --- | --- | --- | --- | --- | --- | --- | --- | --- | --- | --- | --- | --- |
| Overall Quality | 80% **** | 40% ** | 40% ** | 80% **** | 60% *** | 40% ** | 40% ** | 60% *** | 0 | 60% *** | 60% *** | 60% *** | 60% *** | 60% *** | 80% **** |
